# Antibody-Based Therapeutic Interventions for Amyotrophic Lateral Sclerosis: A Systematic Literature Review

**DOI:** 10.3389/fnins.2021.790114

**Published:** 2021-11-29

**Authors:** Amélie Poulin-Brière, Edris Rezaei, Silvia Pozzi

**Affiliations:** ^1^Department of Psychiatry and Neuroscience, Laval University, Quebec, QC, Canada; ^2^Cellular and Molecular Neuroscience Division, CERVO Brain Research Centre, Quebec, QC, Canada

**Keywords:** amyotrophic lateral sclerosis, antibody-based therapy, systematic review, knowledge synthesis, passive immunization

## Abstract

Amyotrophic Lateral Sclerosis (ALS) is a mid-life onset neurodegenerative disease that manifests its symptomatology with motor impairments and cognitive deficits overlapping with Frontotemporal Lobar Degeneration (FTLD). The etiology of ALS remains elusive, with various mechanisms and cellular targets implicated, and no treatment can reverse or stop the progression of the pathology. Therapeutic interventions based on passive immunization are gaining attention for neurodegenerative diseases, and FDA recently approved the first antibody-based approach for Alzheimer's disease. The present systematic review of the literature aims to highlight the efforts made over the past years at developing antibody-based strategies to cure ALS. Thirty-one original research papers have been selected where the therapeutic efficacy of antibodies were investigated and described in patients and animal models of ALS. Antibody-based interventions analyzed, target both extracellular molecules implicated in the pathology and intracellular pathogenic proteins known to drive the disease, such as SOD1, TDP-43 or C9ORF72 repeats expansions. The potentials and limitations of these therapeutic interventions have been described and discussed in the present review.

## Introduction

Amyotrophic lateral sclerosis (ALS) is a devastating neurodegenerative disease that primarily affects motor neurons (MN) of the motor cortex and spinal tract. Ninety percent of ALS cases have sporadic origin (sporadic ALS, sALS), whereas the remaining 10% develop the disease due to inherited gene mutations (familial ALS, fALS). The clinical manifestations of these two groups of ALS are indistinguishable, suggesting a convergence of varying pathways onto one unambiguous outcome, the degeneration of motor neurons and loss of muscles functions. Although several efforts have been made to decipher the pathogenic mechanisms of ALS, its etiology remains elusive, with various mechanisms and cellular targets suggested.

To date, there is no effective treatment for ALS. Patients are currently treated with Riluzole and Edaravone, two drugs approved by the Food and Drug Administration (FDA), that increase the survival up to 14 months (Miller et al., 2012) and slow the disease progression (Abe et al., 2017). Different therapeutic strategies have been tested in animal models with promising results. Kinase inhibitors such as Masitinib (tyrosine kinase inhibitor) or Fasudil (rho kinase inhibitor) increase survival and slow the disease progression in SOD1G93A mice (Takata et al., 2013; Trias et al., 2016). Gene-therapy based strategies are now becoming more available and specifically tested for ALS conditions (Amado and Davidson, 2021). Antisense oligonucleotides (ASO) against SOD1, C9ORF72 repeats, ATXN2 and FUS demonstrate beneficial effects in animal models. Gene editing, using the CRISPR/Cas9 technology, has been implemented for SOD1 and C9ORF72 repeats expansions with promising results in patients-derived cells and animal models. Clinical trials are currently underway to test the efficacy of these approaches in ALS patients (Amado and Davidson, 2021; Yang et al., 2021).

In recent years, there has been increasing interest in the use of monoclonal antibodies to treat neurodegenerative disorders (Freskgård and Urich, 2017; Mortada et al., 2021), with the goal of targeting misfolded intra- or extra-cellular proteins, such as amyloid beta peptide, tau, or alpha-synuclein (Valera et al., 2016). Very recently, the U.S. FDA has approved Aducanumab, a recombinant monoclonal antibody against amyloid beta plaques, for the treatment of Alzheimer's disease patients (Nimmo et al., 2021). Antibodies show a considerable number of advantages when used for therapeutic purposes (Elgundi et al., 2017; Slastnikova et al., 2018; Regazzi et al., 2020). They possess a long half-life, and, due to their nature, they can efficiently target proteins in their physiological state, after post-translational modifications or in a misfolded conformation, with high specificity and affinity. In addition, they can be conjugated to effector molecules and engineered to bind multiple targets. Finally, antibodies can be improved to interact with specific intracellular or extracellular proteins, and can be fragmented to nanobodies for efficient cellular penetration.

Multiple efforts have been made in the past 15 years to develop and test antibodies for therapeutic purposes in ALS. The present review aims to collect and discuss scientific papers where the therapeutic effect of an antibody-based approach was evaluated for ALS patients or models, describing both the potentials of the therapeutic interventions and their limitations.

## Methods

### Research Question and Objectives

In the present review, we aim at providing an overview of the therapeutic interventions based on the delivery of antibodies (passive immunization approaches) that have been developed and tested in patients and/or models of ALS. Here, we summarize the main publications, based on original and experimental data, that proposed therapeutic interventions with a focus on target, antibody used, outcomes and limitations.

### Data Source and Search Strategy

The present review was developed following the PRISMA protocol (Liberati et al., 2009; Moher et al., 2009) with no restrictions on journal or period of publication. The data collection was conducted in May 2021, therefore all papers published after this period were not considered. The search was performed in PubMed, Web of Science, Scopus and Embase databases, using the following main keywords: “amyotrophic lateral sclerosis,” “ALS” and “antibod^*^,” that allowed the inclusion of both “antibody” and “antibodies” wordings.

### Screening Process and Eligibility Criteria

All the publications found were imported in the citation manager Endnote (Endnote, citation manager software, version X7 for Windows, Clarivate Analytics, Philadelphia) for further screening. We first eliminated duplicates, and then performed an “exclusion on format” analysis, aimed at removing all studies that were not original articles based on empirical data, thus only research articles, clinical trials and case reports were further considered. We proceeded to an “exclusion on language” and kept all the studies that were in English, French or Italian as authors could further select and analyze the papers in these languages. Using Endnote we performed an “exclusion on keywords.” Titles, abstracts and keywords were screened for the presence of population keywords: “amyotrophic lateral sclerosis” and “ALS,” keeping only papers focusing on ALS, and the presence of intervention keywords: “therap^*^,” “therapeutic,” “treatment,” “antibod^*^” and “immunoglobulin.” Selected studies were analyzed using the software Covidence (Covidence, Systematic Review Software, Melbourne, retrieved from https://www.covidence.org). Titles and abstracts of manuscripts were fully screened to assess their relevance for our review and excluded if they were not describing a therapeutic intervention based on the development or evaluation of an antibody-based approach. A full-text screening was then performed on the four following criteria of eligibility: nature, finality, method, and availability (Lawarée et al., 2020). Based on nature criteria, all non-empirical publications such as reviews were excluded. Review papers were not included in the final analysis but considered only as key references to understand the overall portrait of the topic analyzed, and to verify the presence of an already published work on the same subject. Studies, where the objective was not to describe or to test a therapeutic intervention based on antibodies, were excluded on finality criteria. Method criteria were used to exclude publications lacking details on the experimental plan and scientific data. Papers not available online or requiring a paid registration not provided by our institution (Université Laval) were excluded based on the availability criteria.

### Data Extraction

Selected papers were compiled in one table (now divided in Tables 1, 2) where the main bibliographic information (first author and year of publication) and the details about the study (targeted protein, type of antibody used, antibody clone, targeted epitope, model used and treatment details) where listed together with observed outcomes and limitations, according to the SALSA framework (Grant and Booth, 2009).

**Table 1:** Summary of the main characteristics of the antibody-based interventions identified in the 31 original articles analyzed in this work.

**Target**	**Antibody**	**Clone**	**Epitope**	**References**
mSOD1	mAb	C4F6	a.a. Asp90-Asp96	Urushitani et al., 2007
	mMAb	D3H5	D3H5: a.a. 24-55	Gros-Louis et al., 2010
		A5C3	A5C3: a.a. 80-118	
	Fab	D3H5		
	HuMAb	HuMAb120c	HuMAb120c: a.a. 36-71	Broering et al., 2013
		HuMAb37L-63	HuMAb37L-63: a.a. 11L-80	
	mMAb	DSE2-3H1	a.a. 125-142	Pokrishevsky et al., 2017
	hMAb	chα-mSOD1	a.a. K76-V82	Maier et al., 2018
	scFv	D3H5	a.a. 24-55	Patel et al., 2014
SOD1	mMAb	α-SOD1a143-153	a.a. 143-153	Lehmann et al., 2020
		α-SOD1 65-72	a.a. 65-72	
	scFv	B1	Not specified	Ghadge et al., 2013
		B12		
	scFv	B1	Not specified	Ghadge et al., 2019
		B12		
	scFv	W20	Not specified	Dong et al., 2018
TDP-43	mMAb	E6	RRM1 domain	Pozzi et al., 2020
	scFv	3B12A	a.a. D247 or the RRM2	Tamaki et al., 2018
	scFv	E652	RRM1 domain	Pozzi et al., 2019
C9ORF72 repeats expansion	mMAb	5F2	Not specified	Zhou et al., 2017
	mMAb	5F2	Not specified	Khosravi et al., 2020
	hAb	α-GA1, α-GP1, α-GA2	Not specified	Nguyen et al., 2020
Nogo-A	HuMAb	Ozanezumab	N-Term of Nogo-A	Meininger et al., 2014
	HuMAb	Ozanezumab	N-Term of Nogo-A	Meininger et al., 2017
	mMAb	GSK577548	N-Term of Nogo-A	Bros-Facer et al., 2014
MuSK	hMAb	#13, #21, #22	Not specified	Cantor et al., 2018
	Chimeric Ab	#13	Not specified	Sengupta-Ghosh et al., 2019
IL-6R	HuAb	Tocilizumab	Not specified	Fiala et al., 2013
	HuAb	Tocilizumab	Not specified	Lam et al., 2016
NRP-1	HuMAb	α-NRP1A	Sema3A docking CUB domains (a1a2) on NRP-1	Venkova et al., 2014
				
Myostatin	MAb	RK35	Not specified	Holzbaur et al., 2006
CD40L	MAb	MR1	Not specified	Lincecum et al., 2010
DR-6	Mab	5D10	Not specified	Huang et al., 2013
IFN-g	MAb	R4-6A2	Not specified	Otsmane et al., 2014
GD1a/GT1b	hAb	rHIgM12	Not specified	Xu et al., 2015
CTGF	hMAb	FG-3019	Not specified	Gonzalez et al., 2018
HMGB1	HuMAb	2G7	a.a. 53-63 (box A of HMGB1)	Lee et al., 2019

*a.a., amino acids; Ab, antibody; h, human; Hu, humanized; m, mouse; MAb, monoclonal antibody; mMAb, mouse monoclonal antibody; mSOD1, misfolded SOD1; scFv, single-chain variable fragment*.

**Table 2:** Summary of the main characteristics of treatments tested in the 31 original articles analyzed in this work.

		**Treatment**					
**Target**	**Antibody**	**Model**	**Stage**	**Delivery, frequency and duration**	**Observed outcomes**	**Observed limitations**	**References**
mSOD1	mAb: C4F6	SOD1G93A mice	Pre-symp. (85 days)	Continuous ICV infusion for 28 days	Delayed body weight loss and motor impairments, increased survival		Urushitani et al., 2007
	mMAb: D3H5, A5C3	SOD1G93A mice (females)	Pre-symp. (65 days) and symp. (95 days)	Continuous ICV infusion for 42 days	D3H5 reduced levels of mSOD1, delayed symptom onset and prolonged lifespan	A5C3 failed to confer protection	Gros-Louis et al., 2010
	Fab fragment: D3H5				Fab fragment extended lifespan		
	HuMAb: HuMAb120c, HuMAb37L-63	SOD1G93A mice (males)	Pre-symp. (65 days)	Continuous IT infusion for 50 days	Increased survival	No evaluation of MN loss, muscle strength, neuroinflammation or SOD1 aggregates	Broering et al., 2013
				IP injections: 3 daily doses at the beginning then once every 7 days			
	mMAb: DSE2-3H1	HEK293FT cells		Ab added in cells medium prior to patient tissues homogenates	Reduced aggregated SOD1 propagation	No studies *in vivo*	Pokrishevsky et al., 2017
	hMAb: chα-mSOD1	SOD1G93A and SOD1G37R mice	Pre-symp. (60 days)	Continuous ICV infusions	Delayed onset, extended survival, reduced SOD1 pathology and MN degeneration, decreased neuroinflammation		Maier et al., 2018
				Weekly IP injections.			
	scFv: derived from D3H5	SOD1G93A mice	Pre-symp. (45 days)	Continuous production after one IT injection of AAV	Delayed disease onset and extension of life span in correlation with scFv titers in the spinal cord. Reduced neuronal stress signals, levels of mSOD1, gliosis and MN loss		Patel et al., 2014
SOD1	mMAb: α-SOD1 a143-153, α-SOD1 a65-72	SOD1G85R mice	Symp. (78 days)	IT inoculation of seed pre-incubation with the antibody	α-SOD1143-153 attenuated transmission of pathogenic aggregation and prolonged the survival	Poor access to intracellular aggregation and insufficient antibody titer in the CNS	Lehmann et al., 2020
				IT inoculation of seed and weekly IP injections till end-stage of the disease		α-SOD1 a65-72 showed adverse effects and increased seed-induced disease	
	scFv: B1, B12	NSC-34 cells		Transfection of heavy and light chain	Decreased SOD1 aggregation, improved cell survival		Ghadge et al., 2013
	scFv: B1, B12	SOD1G93A mice	Neonatal and early-symp. (120 days)	IV infusion of AAV	Decreased MN loss, neuroinflammation and SOD1 burden and aggregation. Increased survival	No significant difference in disease onset	Ghadge et al., 2019
						B12 was toxic for neonatal mice	
	scFv: W20	SOD1G93A mice	Pre-symp.	IN daily administration for 3 weeks	Reduced SOD1 aggregates and total SOD1 levels. Improved motor functions. Reduced weight loss, gliosis and neuroinflammation. Delayed symptoms onset		Dong et al., 2018
TDP-43	mMAb: E6	TDP-43A315T mice	9 months old	IT injections twice a week, for 5 weeks	Decreased TDP-43 mislocalization and reduction of nuclear p65 in MN	Induced a general microglial activation in lumbar spinal cord	Pozzi et al., 2020
		N2A and BV2 cells		In the cell medium	Reduced cytoplasmic TDP-43 by TRIM-21/proteasome pathway		
	scFv: 3B12A	HEK293A cells		Transfection in cells	In cells: Increase cell viability and enhanced TDP-43 aggregate clearance *via* proteasome		Tamaki et al., 2018
		*in utero* electroporation of mutant TDP-43	Embryo stage	Constant production from embryonic stage after scFv plasmid *in utero*	In mice: reduced TDP-43 aggregates in embryonic mouse brain, induced HSP70 transcription and enhanced TDP-43 clearance by promoting protein refolding		
	scFv: VH7Vk9 derived from E652	TDP-43G348C mice and TDP-43A315T mice	Symp. (8 months)	IC or IT single AAV injection	Reduced cognitive and motor deficits in mice		Pozzi et al., 2019
					Reduced TDP-43 proteinopathy and neuroinflammatory		
		HEK293 cells		Transfection	Decreased of total cellular levels of TDP-43 promoting its degradation *via* proteasomal and autophagic pathways		
C9ORF72 repeats expansion	mMAb: 5F2	HEK293 cells, Primary neurons		In the cell medium	Reduced poly-GA levels, inhibited intracellular poly-GA aggregation and blocked the seeding activity of C9orf72 brain extracts		Zhou et al., 2017
	mMAb: 5F2	Primary neurons		In the cell medium	Reduced poly-GA aggregation and transmission, and reduced cytoplasmic levels of TDP-43		Khosravi et al., 2020
	hAb: α-GA1, α-GP1, α-GA2	Female C9-BAC mice	6 weeks of age	Single IP injection	Reduced GA aggregates, improved behavioral deficits, decreased neuroinflammation and neurodegeneration, and increased survival	Anti-GA antibodies more efficient than anti-GP	Nguyen et al., 2020
		HEK293T cells		In the cell medium			
Nogo-A	HuMAb: Ozanezumab	sALS human patients	18–80 years old	IV injections, single dose or two repeated doses, four weeks apart (various dosage).	Treatment was well tolerated.	Did not show changes in functional endpoints. Inclusion criteria and small sample size might have caused bias.	Meininger et al., 2014
	HuMAb: Ozanezumab	sALS human patients	18–80 years old	IV injection every two weeks, for 46 weeks	Treatment was well tolerated.	Did not show changes in functional endpoints nor survival.	Meininger et al., 2017
	mMAb: GSK577548	SOD1G93A mice	Pre-symp. (70 days)	IP weekly injections, until symp. stage (90 days) or end stage (120 days)	Improved muscle innervation, increased muscle strength and motor unit survival, increased MN survival.	Effect was limited to early stages of the disease.	Bros-Facer et al., 2014
MuSK	hMAb: #13, #21, #22	SOD1G93A mice	Symp. (90 days)	Single IP injection, or repeated every 24 days.	Slowed muscle denervation, promoted neuron survival, improved motor system output and extended lifespan.		Cantor et al., 2018
	Chimeric Ab: #13	SOD1G93A mice	Pre-symp. (6 weeks)	IP injections every two weeks for 12 or 16 weeks.	Preserved innervation of the NMJ.	Did not preserve diaphragm function, MN, nor increased survival.	Sengupta-Ghosh et al., 2019
IL-6R	HuAb: Tocilizumab	sALS human patients	Mean age: 55 years old	IV injections every 4 weeks for 4 to 8 months.	Inhibited IL6 signaling and downregulated inflammation.		Fiala et al., 2013
	HuAb: Tocilizumab	Rat cortical neurons exposed to ALS-PBMC supernatant		PBMC supernatant and antibody added to medium.	Inhibited ALS-PBMC supernatant toxicity.		Lam et al., 2016
NRP-1	HuMAb: α-NRP1A	SOD1G93A mice	Pre-symp. (40 days) or advanced stage (90 days)	IP injections twice a week, until end-stage	Temporarily reversed motor functional decline and prolonged the life span, reduced NMJ denervation and attenuated pathologic alterations in ventral roots	No significant efficacy when applied at a late disease stage	Venkova et al., 2014
		NSC-34 cells		In cell medium.	Prevented Sema3A-induced growth cone collapse		
Myostatin	Mab: RK35	SOD1G93A mice and rats	Pre-symp. (28 days)	IP injections weekly until end-stage	Increased skeletal muscle mass and strength in early stages, slowed degenerative changes in skeletal muscle	Did not delay onset nor enhance survival	Holzbaur et al., 2006
						Effects were not observed in advanced stage of the disease	
CD40L	Mab: MR1	SOD1G93A mice	Pre-symp. (50 days)	IP injections weekly until end-stage	Slowed weight loss, delayed disease onset, extended survival, reduced neuroinflammation and reduced MN loss		Lincecum et al., 2010
DR-6	Mab: 5D10	SOD1G93A mice	Pre-symp. (42 days)	IP injections twice a week, until end stage (140 days)	Decreased gliosis, increases survival of MN and oligodendrocytes, protected NMJ, improved motor function and decreased pNfH levels in serum		Huang et al., 2013
			Motor neurons	In the cell medium after stimuli	Prevent challenger-induced death		
IFN-γ	MAb: R4-6A2	SOD1G93A mice	Early-onset (13 weeks)	Continuous ICV infusion	Increase MN survival	Did not increase survival. Therapeutic benefit obtained only during antibody delivery	Otsmane et al., 2014
		Motor neurons		In cells medium	Reduced IFN-g-induced death		
GD1a/GT1b	hAb: rHIgM12	SOD1G93A, SOD1G86R mice	Pre-symp. (60 days)	Single IP injection	Increased survival, delayed the onset of symptoms preserved MN from death	Multiple doses were ineffective due to anti-human response	Xu et al., 2015
		Primary neurons		In cells medium	Promoted cytoskeleton dynamics		
CTGF	hMAb: FG-3019	SOD1G93A mice (males)	Pre-symp. (8 weeks)	IP injections trice a week for 2 months	Improved muscle function and locomotor capacity. Reduced pathological events in skeletal muscle. Improved NMJ innervation.		Gonzalez et al., 2018
HMGB1	HuMAb: 2G7	SOD1G93A mice (females)	Pre-onset (35 days) or post-onset (70 days)	IP injections weekly until end-stage	Pre-onset: Transiently improved muscle strength and reduced key pro-inflammatory genes	Overall, had limited efficacy and did not extend survival time	Lee et al., 2019
						Post-onset: No effect	

*Ab, antibody; AAV, adenoassociated virus; h, human; Hu, humanized; ICV, intracerebroventricular; IP, intraperitoneal; IT, intrathecal; IV, intravenous; IN, intranasal; m, mouse; MAb, monoclonal antibody; mMAb, mouse monoclonal antobody; MN, motor neurons; mSOD1, misfolded SOD1; NMJ, neuromuscular junction; Pre-symp., presymptomatic; sALS, sporadic ALS; scFv, single-chain variable fragment; Symp., symptomatic*.

### Data Analysis

A thematic summary technique was used for data synthesis. We defined “targeted protein” as a specific thematic category to classify the selected publications and grouped single manuscript studies under the “other targeted proteins” category. We revised and discussed the outcomes of the studies under each category, evaluating the type of antibody employed, the specific epitope bound, and the models used for the study.

## Results

### Description of the Selected Papers and Identification of Thematic Categories

The systematic review conduced for antibody-based therapeutic interventions for ALS resulted in the final selection of 31 papers listed in Tables 1, 2.

**Figure 1:**
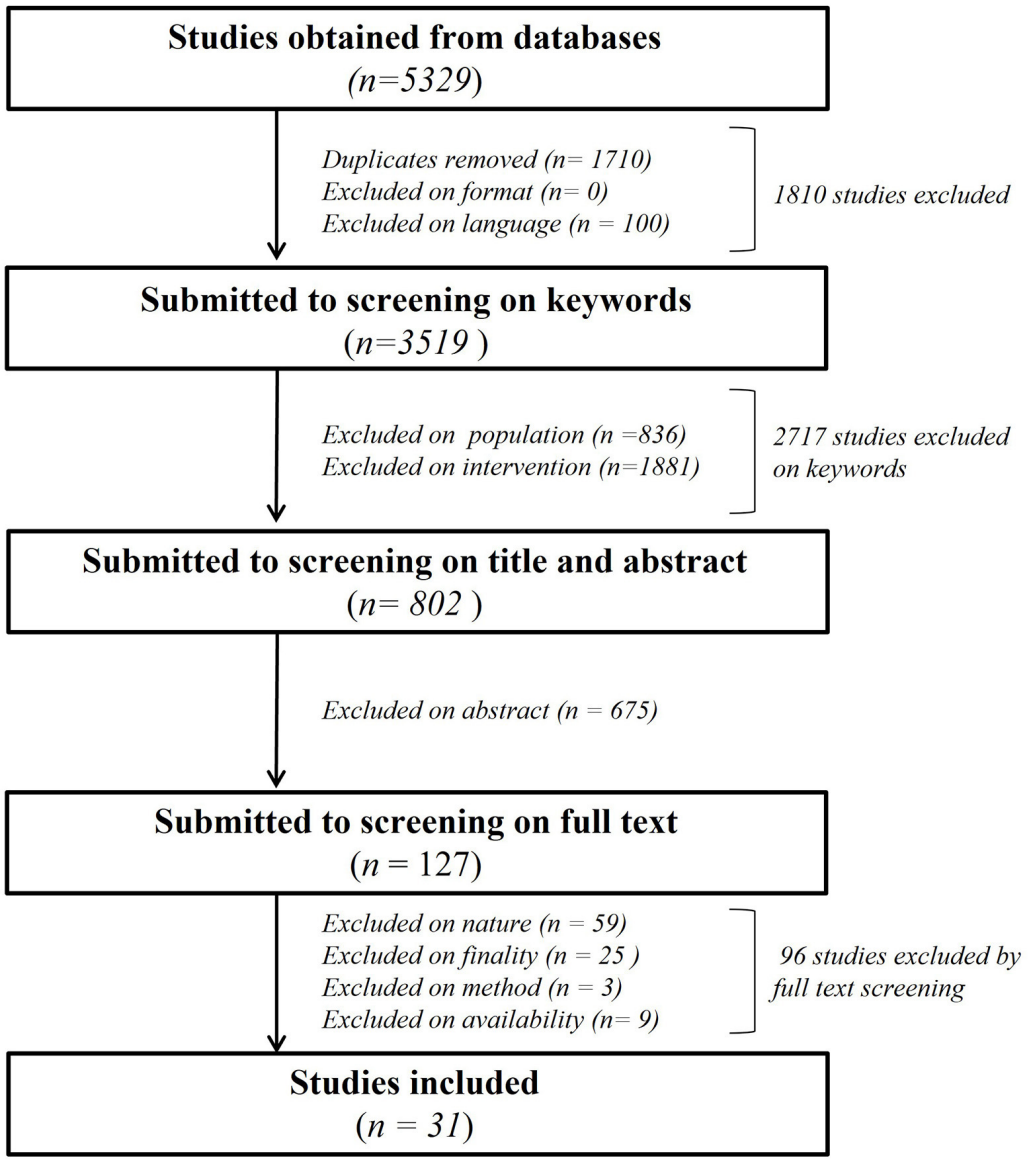
Flowchart of the systematic literature analysis describing all the steps of the manuscript's selection leading to the 31 analyzed paper.

As shown in Figure 1, we primarily found a total of 5,329 publications to screen. 1,710 papers were excluded as duplicates, none after screening for “exclusion on format” criteria and 100 based on “exclusion on language.” A total of 2,717 publications were eliminated after “exclusion on intervention keyworks” in title, abstract and keywords session. *N* = 836 were eliminated for “population” and *n* = 1,881 for “intervention” keywords, leaving 802 articles for further analysis with Covidence software. The title and abstract of the remaining papers were fully screened (Supplementary Table 1), leading to exclusion of *n* = 675 manuscripts of which the subject was not relevant for our review despite the presence of keywords, leaving 127 papers for further screening (Supplementary Table 2). The final full text analysis based on nature, finality method and availability criteria led to the exclusion of 96 total studies. *N* = 59 studies were excluded based on nature, *n* = 25 based on finality, *n* = 3 based on method and *n* = 9 based on availability of a full text to analyze, leaving a total of 31 studies for the present review.

**Figure 2:**
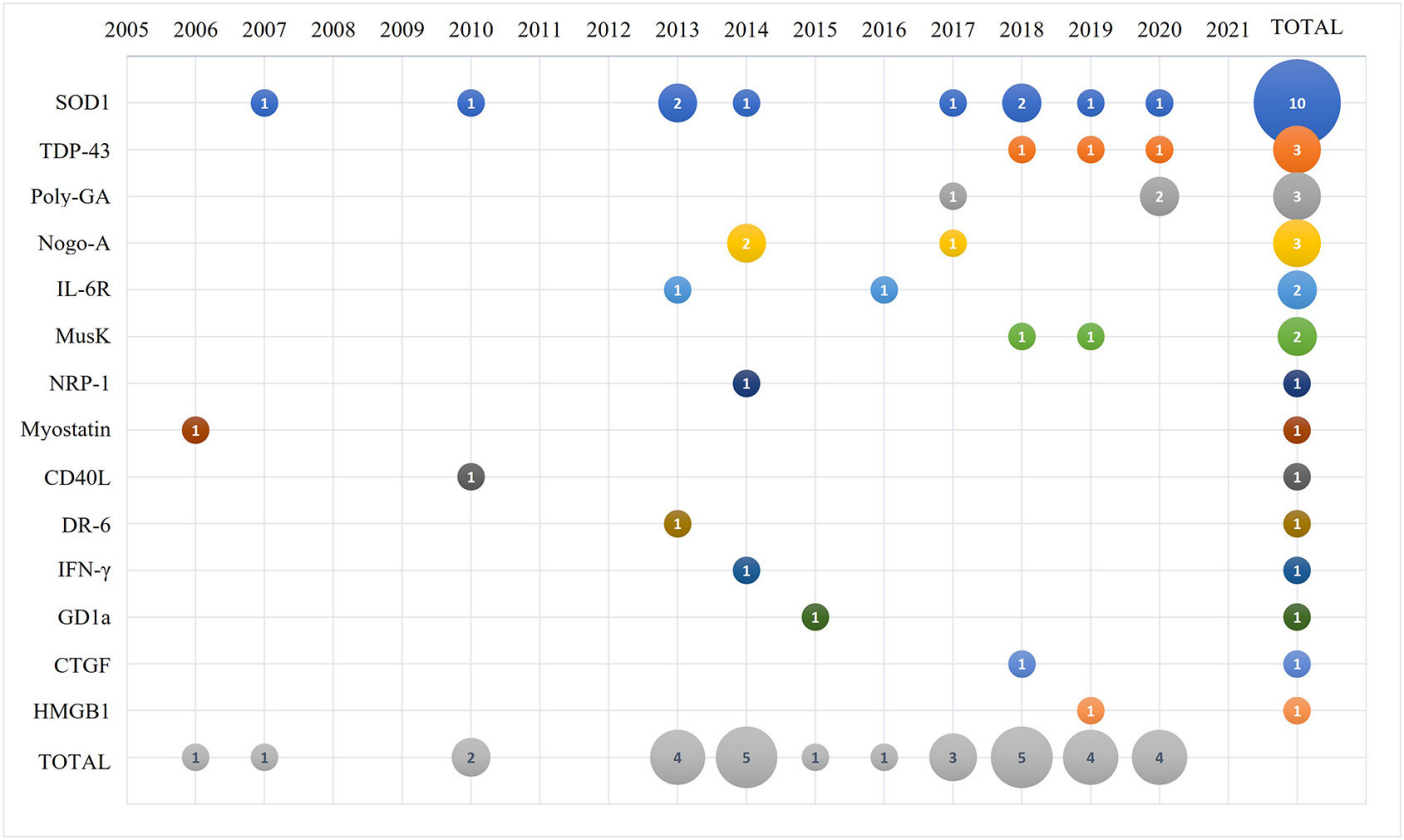
Bubble plot of the 31 selected and analyzed papers describing the number of manuscripts published (number in the bubble) per selected target (y-axis) and time (x-axis).

The selected papers were analyzed by year of publication as shown in Figure 2. The first paper describing an antibody-based therapy for ALS targeted myostatin and was published in 2006, followed by a paper targeting SOD1 that was published at the beginning of 2007. The majority of the remaining selected papers were published between 2010 and 2020. We identified the categories for the thematic summary and applied them to the selected papers. As summarized in Figure 2, SOD1 is presently the main target of antibody-based approaches described in 10 papers; antibodies against TDP-43 and C9ORF72 repeats were discussed in 3 papers each; 3 papers described antibodies against Nogo-A; 2 papers described antibodies against IL-6 receptor and Musk; and a total of 8 manuscripts targeted respectively NRP-1, myostatin, CD40 ligand, DR-6 receptor, INF-γ, GD1a, CTGF and HMGB1 that were then grouped in the thematic category of “other targeted proteins.”

### Antibody-Based Interventions for SOD1

Superoxide dismutase 1 (SOD1) have been unequivocally implicated in ALS. SOD1 mutation accounts for 2–5% of ALS cases (Gros-Louis et al., 2010). Moreover, SOD1 intracellular aggregation, which causes specific degeneration of motor neurons (Gurney et al., 1994), has been observed in sporadic ALS cases without SOD1 mutation (Bosco et al., 2010; Forsberg et al., 2010; Guareschi et al., 2012; Pokrishevsky et al., 2012; Paré et al., 2018). From the 31 studies included, 10 studies were focused on the protein SOD1. These studies were published between 2007 and 2020. Five of them used full-length monoclonal antibodies (MAb), three with mouse and two with human origin. One used a full-length antibody purified from antisera of actively immunized mice, whereas the other four studies used single-chain variable fragments (scFv).

#### Full Length Antibodies Against SOD1

The first passive immunization approach against misfolded SOD1 was studied and proposed by Urushitani et al. (2007). The aim of the study was to test active and passive immunization protocols aimed at reducing the burden of extracellular SOD1 mutants in the nervous tissue of mouse models of ALS. An antibody specific for human misfolded SOD1 (a.a. Asp90–Asp96), named C4F6, was purified from C57BL/6 mice inoculated with recombinant human G93ASOD1 and administered to SOD1G93A mice by intraventricular infusion for 28 days starting from the pre-symptomatic stage (85 days of age). Disease progression was monitored using a temporal profile of the body weight and hindlimb reflex score and the passive immunization approach demonstrated to significantly delay body weight loss, hindlimb reflex impairment, and to increase the life span of treated mice. By highlighting the significant effect of passive immunization vs. active immunization, this paper opened the door for antibody-based approaches against SOD1 for ALS treatment (Urushitani et al., 2007).

In 2010, Gros-Louis et al. investigated passive immunization of mutant SOD1G93A female mice with D3H5 or A5C3, two mMAb targeting misfolded SOD1 (mSOD1) (Gros-Louis et al., 2010). The antibodies were obtained from supernatant of hybridoma cell lines derived from mice injected with the metal free apo-form of recombinant SOD1 with G93A mutation as antigen. A5C3 antibody targets amino acids (a.a.) 80-118, while D3H5 targets a.a. 24-55 (Rotunno and Bosco, 2013). After intracerebroventricular (ICV) infusion for 42 days, D3H5 antibody succeeded in delaying symptoms onset and mortality of ALS mice and in reducing mSOD1 levels by 23% in spinal cord of treated mice compared with a group infused with control mouse IgG. The treatment showed interesting, although not significant, reduction in motor neuron loss, attenuation of astrocytosis, and increased number of innervated neuromuscular junctions (NMJ). Authors also tested the Fab fragment of D3H5, which demonstrated to confer some protection by extending the lifespan by 6 days. On the other hand, A5C3 failed to confer any protection (Gros-Louis et al., 2010).

Eleven humanized MAbs directed against human SOD1, five of which binding only to unfolded or misfolded hSOD1, were developed by Broering et al. in 2013 (Broering et al., 2013). During exploratory experiments, male mice expressing SOD1G93A were treated with the antibodies in the cerebrospinal fluid (CSF) by lumbar intrathecal (IT) injections with osmotic pump for 50 days, starting from a pre-symptomatic stage of the disease (65 days of age). Although not significant, due to small group size, antibodies showed no toxicity and tended to increase survival in SOD1G93A mice. Experiments were then repeated in a larger cohort of mice with the two antibodies that yielded the best results in terms of survival, i.e., HuMAb120c and HuMAb37L-63, confirming their therapeutic effect. These two antibodies recognize linear epitopes and bind to a.a. 36-71 present on the zinc-binding loop (Ab120c) and to 11L-80 epitope (Ab37L-63), not accessible in properly folded hSOD1. HuMAb37L-63 was also tested by intraperitoneal (IP) administration. This delivery method yielded higher levels of antibody in the nervous tissue but same survival time as IT injections. No further analyses on motor neuron loss, muscle strength, neuroinflammation or SOD1 aggregates were performed in this study (Broering et al., 2013).

Few years later, Pokrieshevsky et al. conducted a study using a mouse monoclonal antibody (mMAb) specific to misfolded SOD1 (DSE2-3H1), binding to a.a. 125-142 of extended electrostatic loop of full-length SOD1 protein, demonstrated to confer prion-like properties to the protein (Rotunno and Bosco, 2013; Pokrishevsky et al., 2017). In HEK293FT cells transfected with chimeric SOD1-GFP protein and exposed to hSOD1-fALS spinal cord tissue homogenates, the group demonstrated the ability of misfolded SOD1, present in the fALS tissues, to trigger the aggregation of the reporter protein. Interestingly, the treatment with DSE2-3H1antibody for 30–45 min prior to the exposure of tissues homogenates successfully attenuated the aggregation of SOD1-GFP protein (Pokrishevsky et al., 2017).

In 2018, Maier et al. developed a recombinant human monoclonal antibody (hMAb), specific to human misfolded SOD1 (Maier et al., 2018) and, to avoid mouse anti-human responses upon chronic use, a chimeric version was used in ALS mice models. The antibody was designed to specifically target a.a. K76-V82 in the IV loop of native SOD1. ICV infusion with minipumps from a pre-symptomatic stage (60 days of age) until the end stage of disease ameliorated motor symptoms and reduced SOD1 pathology in SOD1G93A mice. Gait abnormalities were significantly improved, body weight loss was attenuated, and median survival increased compared to vehicle-treated control littermates. Histological analysis of spinal cords revealed a trend toward a decrease in neuroinflammation and a significant reduction in SOD1 aggregate amount by 51%. When administered IP as a single dose, the antibody was able to penetrate the central nervous system (CNS) and bind to misfolded SOD1. It improved the survival of SOD1G93A mice and their motor performance, even at low doses. It led to 66% reduction in misfolded SOD1 and 25% reduction in SOD1 aggregates together with a reduction of 37% in microgliosis and 43% in astrogliosis. In slowly progressing SOD1G37R mice, when injected IP, the chimeric antibody delayed disease onset, ameliorated motor impairments and increased survival. Mice treated with chimeric α-mSOD1 displayed higher muscular strength, with 20% increase in weight of the hindlimb muscles, and preservation of 50% of motor neurons (Maier et al., 2018).

More recently, Lehmann et al. investigated the effect of disordered SOD1-selective antibodies on the SOD1 transmission of aggregation properties. In their model, intrathecal inoculation of hSOD1 aggregates isolated from the CNS of ALS mice model exacerbated pathology and SOD1 aggregation in SOD1G85R mice (Lehmann et al., 2020). Authors developed two mMAbs specific to epitopes exposed only in aggregated forms of human SOD1 (hSOD1), i.e., a.a. 143-153 of C-terminus of hSOD1 (α-SOD1143-153) and a.a. 65-72 (α-SOD165-72). Antibodies were pre-incubated with the aggregate strains and further injected into mice, or directly tested by weekly IP injections before inoculation of aggregate strains. In both cases, Ab administration or Ab-preincubated strains, were delivered from early symptomatic stage (78 days of age) until end-stage of the disease. Both pre-incubation with aggregates and IP injections of α-SOD1143-153 attenuated transmission of pathogenic aggregation and prolonged the survival of transgenic mice by up to 47%. α-SOD165-72 antibody, which displayed lower affinity for hSOD1, showed adverse effects and aggravated seed-induced ALS-like disease. Moreover, α-SOD1143-153 was found in association with aggregated hSOD1 in spinal cord homogenates. However, the administration of antibodies in non-inoculated hSOD1G85R mice did not improve survival nor mitigated spontaneously evolving aggregation, suggesting that the antibody effect is specific to seed-induced ALS-like disease (Lehmann et al., 2020).

#### Nanobodies Against SOD1

The first nanobodies against SOD1 were generated by Ghadge et al. (2013), who developed two human-derived single chain antibodies (scFvs) named B1 and B12, binding to all three WT, G93A and A4V SOD1 forms (Ghadge et al., 2013). NSC-34 cells were co-transfected with the scFv antibody and A4VSOD1-YFP to induce SOD1 aggregation. After 48 h of A4VSOD1-YFP transfection, ~24% of YFP expressing cells had bright focal punctuate areas of fluorescence that were significantly decreased following co-transfection with B1 or B12 scFv. Clones B1 and B12 also improved cell survival following expression of either A4V or G93A mutations compared to control groups with empty vector or a negative control scFv (Ghadge et al., 2013). In 2019, the same group subcloned B1 and B12 into an AAV9 virus and injected it intravenously (IV) in neonatal and early symptomatic (120 days old) SOD1G93A mice. B1 and B12 decreased motor neuron loss, microgliosis, astrogliosis, and SOD1 amount and aggregation, as shown by reduction of percentage of anti-SOD1 positive area in spinal cord sections. No significant difference in the disease onset was observed, but the α-SOD1 treatment significantly increased survival compared to control mice, with the longest survival measured after AAV:scFvB1 delivery into neonatal SOD1G93A mice. On the contrary, AAV:scFvB12 was toxic for neonatal mice (Ghadge et al., 2019).

Concomitantly to the first single chain antibody, Patel et al. produced in 2014 a scFv antibody derived from the monoclonal antibody D3H5, previously tested by Gros-Louis et al. (2010) (Patel et al., 2014). After a single intrathecal injection of AAV2/1:D3H5scFv in young SOD1G93A mice (45 days of age), AAV-mediated expression of the scFv delayed disease onset and extended life span by up to 28%. Mice survival was directly correlated with the amount of scFv antibody present in the spinal cord. The treatment attenuated neuronal stress pathways such as GAP-43 and ATF3 upregulation, reduced gliosis and levels of misfolded SOD1 in the spinal cord of SOD1G93A mice as well as decreased motor neuron loss (Patel et al., 2014).

Finally, in 2018, Dong et al. produced a human scFv antibody, named W20, targeting toxic SOD1 oligomers, which was delivered intranasally (IN) every day for 3 weeks in SOD1G93A mice before the onset of symptoms (Dong et al., 2018). Authors observed a significant reduction of SOD1 aggregates in the brain stem, and reduced SOD1 levels in the spinal cord lysates of SOD1G93A mice. Oligomeric forms of the protein were reduced in W20-treated mice showing a significant 37.6 and 22.3% of reductions in fibrillar SOD1 and prefibrillar oligomers respectively, compared to vehicle-treated SOD1G93A mice. Treated mice displayed improved rotarod performance, significantly increased muscle strength, reduced hindlimb clasping, reduced weight loss, and markedly reduced gliosis and neuroinflammation, together with a slight delay in symptoms onset (Dong et al., 2018).

### Antibody-Based Interventions for TDP-43

TDP-43 proteinopathy is a hallmark of ALS (Neumann et al., 2006), characterized by cytoplasmic mislocalization and aggregation of the TAR DNA binding protein 43 (TDP-43), which loses its physiological properties, leading to neuronal death. Out of the 31 papers included in the analysis, 3 studies targeted TDP-43 protein. These studies were published between 2018 and 2020. One of them used a monoclonal full-length antibody (MAb), whereas the other two studies used single chain antibodies (scFvs).

#### Full Length Antibodies Against TDP-43

Pozzi et al. produced 8 monoclonal antibodies directed mainly against the RNA recognition motif 1 (RRM1) domain of TDP-43 (Pozzi et al., 2019), known to be sensitive to TDP-43 proteinopathy (Chang et al., 2013; Shodai et al., 2013) and involved in the interaction between TDP-43 and p65 NFκB (Swarup et al., 2011). From those 8 clones, only three of them, named C10, G8 and E6, were able to clearly detect TDP-43 with a visible preference for the cytoplasmic protein. Among the three full-length monoclonal antibodies, the E6 clone was the most effective at interfering with the binding of TDP-43 to p65 (Pozzi et al., 2019). Authors tested the E6 full-length antibody both *in vitro* and *in vivo*, where E6 showed no toxicity (Pozzi et al., 2020). In immortalized neuronal cells, E6 reduced levels of cytoplasmic TDP-43 by 33% compared to control antibody, inducing the TRIM-21 and the lysosome-dependent mechanisms of degradation. E6 full-length antibody also reduced NFκB activation in neuronal cells and in microglial cells challenged with LPS, due to either a reduction of the cytoplasmic TDP-43 or the inhibition of TDP-43/p65 interaction (Pozzi et al., 2020). Repeated intrathecal injections of E6 antibody showed to be safe and feasible for symptomatic TDP-43A315T mice, and the antibody demonstrated a broad diffusion throughout the length of the spinal cord together with motor neuron and microglia uptake. Five-weeks of repeated injections of E6 full-length antibody in TDP-43A315T mice resulted in mitigation of cytoplasmic TDP-43 mislocalization and reduction of nuclear p65 in motor neurons; however, E6 treatment induced a general microglial activation in lumbar spinal cord, suggested to be associated with the Fc domain of the antibody (Pozzi et al., 2020).

#### Nanobodies Against TDP-43

In 2018, Tamaki et al. produced two nanobodies derived from 3B12A MAb directed against the a.a. D247 of the RRM2 domain masked in the physiological state (Tamaki et al., 2018). Authors showed a specific binding of the scFv antibodies to the mislocalized and aggregated form of the protein. The two produced scFvs contained a PEST sequence, which induces proteasome-mediated proteolysis, and were genetically engineered with a chaperone-mediated autophagy (CMA) sequence or a proteasome localization signal (CL1) to enhance their degradation. In transfected HEK293A cell, proteasome-mediated degradation of aggregated TDP-43 was accelerated, enhancing TDP-43 aggregates clearance and cell viability. TDP-43 aggregates were also reduced in brains of an embryonic mouse model where the plasmids encoding for the scFv antibodies were *in utero* electroporated together with mutant TDP-43 plasmids to induce the pathology. Authors correlated the neuroprotection observed in mice to the transcriptional induction of HSP70, suggesting that HSP70 was involved in TDP-43 clearance by promoting protein refolding (Tamaki et al., 2018).

Few months later, Pozzi et al. produced two scFvs antibodies derived from E6 MAb (Pozzi et al., 2020), named VH1Vk9 and VH7Vk9, binding to the RRM1 domain of TDP-43 protein (Pozzi et al., 2019). The scFvs were designed with a secretion signal peptide, which enabled them to target distant cells. ScFv antibodies transfected in HEK293 cells induced a decrease in total cellular levels of TDP-43 by enhancing the polyubiquitination signaling for proteasomal and autophagic degradation. In the same cells, VH1Vk9 and VH7Vk9 reduced TDP-43 mislocalization and aggregation induced by ethacrynic acid (EA) treatment, a model of TDP-43 proteinopathy. Both VH1Vk9 and VH7Vk9 were able to disrupt the protein-protein interaction between TDP-43 and p65 and to reduce LPS-induced activation of microglial cells. As VH7Vk9 was more efficient than VH1Vk9 in reducing NFkB activity and TDP-43 proteinopathy, *in vivo* experiments were conducted by delivering the selected scFv through a viral vector. AAV2/9:VH7Vk9 expression reduced the LPS-induced microglial activation by almost 50% *in vivo*. In symptomatic TDP-43G348C mice, a single intracortical (IC) injection of AAV2/9:VH7Vk9 mitigated cognitive impairment, neuroinflammation and TDP-43 pathology as early as 2 months after injection. Moreover, in symptomatic TDP-43A315T mice, injected IT with AAV2/9:VH7Vk9, the VH7Vk9 scFv antibody improved motor performance and neuromuscular junction innervation, together with reduced neuroinflammation and TDP-43 cytoplasmic accumulation (Pozzi et al., 2019).

### Antibody-Based Interventions for C9ORF72 Repeats Expansion

In 2011, a hexanucleotide GGGGCC (G4C2)n repeat expansion in the C9orf72 gene was found to be implicated in frontotemporal lobar degeneration and amyotrophic lateral sclerosis as the most common known genetic cause (DeJesus-Hernandez et al., 2011; Renton et al., 2011; Gijselinck et al., 2012). These repeats are transcribed into repetitive RNA, which forms sense and antisense RNA (Ash et al., 2013). Despite being within a non-coding region of C9orf72, these repetitive RNAs are translated into five aggregating dipeptide repeat proteins (DPRs) named poly-GA, poly-GP, poly-PA, and poly-PR, forming neuronal inclusions, and sequestering several RNA binding proteins (Mori et al., 2013). Out of 31 studies included, 3 studies focused on the poly-GA protein. These studies were published between 2017 and 2020. Two of them used a mouse monoclonal full-length antibody, whereas the last one used a human recombinant full-length antibody.

In 2017, Zhou et al. studied the spread of intraneuronal pathology of dipeptide repeat proteins (DPRs) and investigated the potential of an antibody therapy to inhibit this phenomenon (Zhou et al., 2017). First, using co-culture systems of primary or immortalized cells, they demonstrated that poly-GA, poly-GP and poly-PA repeat species can be transmitted between cells and cause seed aggregation in recipient cells. They also observed that exposing cells to brain extracts from ALS patients with C9orf72 repeat expansion increased poly-GA, -GR and -GP levels and aggregation, suggesting a vicious cycle. In a second set of experiments, authors tested a mouse monoclonal IgG2A antibody against poly-GA DPRs, named 5F2, produced through immunizing mice with insoluble recombinant poly-GA repeats (Mackenzie et al., 2013). In GA175GFP-transfected HEK293 cells, 3-day treatment with 5F2 anti-GA antibody was able to reduce poly-GA levels and aggregation compared to isotype control treated cells. The effect was confirmed in primary neurons treated for 6 days with 1mg/ml antibody. Finally, preincubation of C9orf72 patients brain extracts with the antibody prevented the seeding of aggregates in (G4C2)80-expressing HEK293 cells (Zhou et al., 2017).

Few years later, Khosravi et al. used co-culture systems of primary neurons to demonstrate that poly-GA aggregation can induce TDP-43 cytoplasmic mislocalization and aggregation in poly-GA expressing cells and in neighboring cells by inhibition of proteasome function (Khosravi et al., 2020). Treatment with the 5F2 anti-GA antibody ameliorated the TDP-43 pathology in both donor and recipient cells. Authors corroborated these results by immunodepleting poly-GA with 5F2 antibody from media of DRPs-expressing cells, establishing that immunodepletion prevented poly-GA uptake in recipient cells and thus reduced TDP-43 pathology compared to depletion with control IgG (Khosravi et al., 2020).

In 2020, Nguyen et al. purified three high affinity human recombinant antibodies against GA and GP C9orf72 RAN proteins, respectively named α-GA1, α-GP1, and α-GA2 (Nguyen et al., 2020). Antibodies were previously obtained from libraries of memory B cells of healthy individuals (Sevigny et al., 2016). The antibodies were able to recognize aggregated GA or GP RAN proteins in both HEK293 cells and C9-BAC mouse tissues, a mouse model of ALS/FTLD containing 500–750 GA-repeats (Liu et al., 2016), as well as in human cerebellar samples with C9orf72 expansions. In C9orf72 patient-derived motor neurons (iMNs), treatment with α-GA1 and α-GA2, but not α-GP1 nor control antibody, reduced GA levels. To understand the mechanisms involved in the increased poly-GA clearance, authors conducted a proximity ligation assay (PLA), demonstrating that TRIM21 protein interacts with the Fc region of α-GA1 enhancing the clearance of poly-GA/anti-GA1 complex. Moreover, they showed that treatment with α-GA1 antibody released proteosomal and autophagy proteins sequestered in poly-GA aggregates, resulting in a rescue of proteasomal activity and increased protein clearance. Anti-GA1, but not anti-GA2 nor anti-GP1, was able to rescue cultured cells from DRPs induced toxicity, suggesting a therapeutic potential for α-GA1. In C9-BAC mice, α-GA1, α-GA2, α-GP2 (30 mg/kg) or PBS was administered by weekly IP injections from 6 to 43 weeks of age. Murine chimeric derivatives were used to avoid mouse anti-human responses. The three antibodies were able to penetrate the CNS and reduced both soluble and aggregated RAN proteins. Both anti-GA antibodies improved survival of C9-BAC mice and reduced gait abnormalities as well as anxiety-like behavior, whereas the α-GP1 antibody did not. Authors hypothesized that a higher reduction of poly-GP was needed for behavioral improvements. Anti-GA1 treatment, which showed higher efficacy in rescuing the phenotype, probably due to its increased affinity for longer GA-repeats (60 and 120), was also able to significantly reduce inflammation (astrocytosis) in motor cortex, and to increase neuronal survival in spinal cord. Interestingly, using an aglycosylated α-GA1 antibody, authors demonstrated, both in cells and mice, that a fully glycosylated Fc region of the α-GA1 antibody is required for α-GA1 cellular uptake and for its therapeutic effects such as reduction of GA-aggregates, neurons preservation, and improvement of behavioral performance (Nguyen et al., 2020).

### Antibody-Based Interventions for the Neurite Outgrowth Inhibitor A

Nogo is a myelin-associated neurite outgrowth inhibitor in the CNS (Caroni and Schwab, 1988), which has three protein products: Nogo-A (1,163 amino acids), Nogo-B (360 amino acids) and Nogo-C (199 amino acids) (Chen et al., 2000; Prinjha et al., 2000). Nogo-A was shown to increase in SOD1G86R transgenic mice at the pre-symptomatic stage, while Nogo-C levels were decreased (Dupuis et al., 2002; Jokic et al., 2006). Moreover, altered expression of Nogo proteins in the post-mortem and biopsy samples from diagnosed ALS patients (brachial plexus nerve and deltoid muscle) was reported (Jokic et al., 2006). Nogo-A expression has been linked to axonal denervation (Jokic et al., 2006). Knock-out of Nogo-A in ALS mice increased the number of healthy motor neurons in soleus muscle and the mice survival, whereas overexpression of Nogo-A in muscles gave rise to the disassembly of the neuromuscular junction (Jokic et al., 2006). Out of 31 studies included, 3 studies focused on the Nogo-A protein. These studies were published between 2014 and 2017. One study used a mouse antibody whereas the other two used a humanized antibody.

Ozanezumab (GSK1223249), a humanized monoclonal antibody (HuMAb) targeting the N- terminus of Nogo A was generated by Schnell and Schwab by immunization of mice in 1990 (Schnell and Schwab, 1990). The Fc portion of the antibody includes two amino acid substitutions at positions 235 and 237, taking away the ability of monoclonal antibodies to recruit immune effector cells and complement. This humanized anti-NogoA antibody was first used in 2014 by Meininger et al. for a phase I study in sporadic ALS patients where safety, pharmacokinetics (PK), and functional effects of Ozanezumab were assessed (Meininger et al., 2014). The trial was performed as a randomized study, in which Ozanezumab was compared to placebo in a double-blind manner. Two groups of patients received respectively (1) a single dose (SD) of Ozanezumab (0.01, 0.1, 1, 5, or 15 mg/kg) or (2) two repeated doses (RD) of Ozanezumab (0.5, 2.5, or 15 mg/kg). In both cases the antibody was administered intravenously (IV). In total, 18 patients (18–80 years of age) received placebo, and 53 patients (age matched) received Ozanezumab. A clear co-localization between Nogo-A and Ozanezumab was observed in skeletal muscle biopsies by IHC staining and the percentage of co-localization correlated with the amount of Ozanezumab injected. Ozanezumab was well tolerated both as SD (0.01–15 mg/kg) and RD (0.5–15 mg/kg). No adverse events (AEs) were reported with increasing doses of Ozanezumab although the incidence of AEs was numerically higher in patients receiving a repeated injection of the two higher doses, but the proportion of AEs and serious adverse events (SAEs) was similar between treated and placebo groups. Generally, SAEs and deaths were not related to the treatment, but doubts were raised for one cardiac non-serious AE (mild sinus tachycardia). Although the study was not designed to obtain relevant observations in functional endpoints, a positive trend in some clinical measurements such as ALS Functional Rating Scale (ALSFRS-R), slow vital capacity (SVC), and manual muscle testing (MMT) was observed with the highest dose (Meininger et al., 2014).

The results of this study allowed the authors to start a Phase II randomized and double-blinded clinical trial, aimed at investigating Ozanezumab efficacy and safety in both familial and sporadic ALS patients (Meininger et al., 2017), who were allowed to continue taking riluzole during the study. Patients were divided into two groups that received either placebo (*n* = 151) or 15 mg/kg of Ozanezumab (*n* = 152) delivered intravenously every 2 weeks for 46 weeks. Assessments were performed at 48 and 60 weeks after treatment. The study was a combined analysis of the ALS Functional Rating Scale-Revised (ALSFRS-R) and overall survival. Even though Ozanezumab was generally well tolerated, and that reported AEs were similar between treatment and placebo groups, no difference was observed in scores nor survival rates at 48 weeks. No efficacy was therefore observed for Ozanezumab, suggesting a poor reliability of anti-Nogo-A treatments for ALS patients (Meininger et al., 2017).

In 2014, concomitantly with the first clinical trial of Ozanezumab, Bros-Facer et al. studied the effect of an anti-Nogo-A antibody on the disease phenotype and progression in the SOD1G93A mice (Bros-Facer et al., 2014). In this study, a murine antibody (GSK577548: the murine parental antibody for Ozanezumab) was generated against Nogo-A. Authors treated SOD1G93A mice by weekly IP injections starting from 70 days of age (pre-symptomatic stage) until 90 (late symptomatic stage) or 120 days of age (near to end-stage). After confirming the presence of the antibody in muscles and its co-localization with Nogo-A, authors observed a reduction of Nogo-A levels, as well as CHRNA1 and MuSK, two markers of neuromuscular dysfunction, in 90 days old anti-Nogo-A treated mice compared to control group. No differences in expression were observed between the groups at 120 days. The innervation of two muscles (slow-twitch soleus muscle and fast-twitch extensor digitorum longus (EDL) muscle) was analyzed by staining for silver cholinesterase showing a clear decrease in denervation in both muscles of antibody-treated SOD1G93A mice at 90 days of age. This improvement in innervation led to an increased muscle force and motor unit survival. Functional physiological analysis of the muscle contractile ability and staining for succinate dehydrogenase (SDH), an oxidative enzyme that increases in fast twitch fibers during disease progression, showed that treatment with the antibody prevented pathological alterations. Similarly, Nissl staining of lumbar spinal cord sections showed 21% increase in motor neuron survival of treated mice. Although encouraging results were observed in treated mice, improvements were mainly observed at 90 days of age and not maintained at end-stage of the disease (120 days of age), concluding that treatment with anti-Nogo-A antibody can significantly improve neuromuscular function only in early disease stages of SOD1G93A mouse model (Bros-Facer et al., 2014).

### Antibody-Based Interventions for the Muscle-Specific Kinase

In ALS animal models, loss of neuromuscular synapses occurs prior to the loss of motor neurons (Alhindi et al., 2021). Although defects in the MuSK signaling pathway are not associated with ALS, studies show that overexpression of MuSK stabilizes neuromuscular synapses in SOD1G93A ALS mouse model, preventing denervation and slowing down motor dysfunction (Pérez-García and Burden, 2012). Moreover, stimulation of the MuSK receptor tyrosine-kinase activity with Agrin leads to anchoring and enhanced expression of critical post-synaptic proteins (Zhang et al., 2011; Burden et al., 2013). Out of 31 studies included, two studies focused on the MuSK protein. These studies were published between 2018 and 2019. Both used a humanized full-length antibody.

In 1997, 21 single-chain antibodies were isolated by phage display with the aim to modulate MuSk functions. Four of these scFvs, named #13, #21, #22 and #2, were highly specific for MuSk. Among them, #13 and #22 were able to activate the kinase and induce AChRs clustering in the cultured myotube cell lines (Xie et al., 1997). In 2018 Cantor et al. generated human IgG1 antibodies from single chain antibodies #21, #13 and #22 that recognize mouse MuSK (Cantor et al., 2018) with the aim to determine whether increasing MuSK activity with a pharmacological approach would preserve neuromuscular synapses in SOD1G93A mice and slow down the loss of motor innervation. Unlike agrin, the agonist antibodies stimulated MuSK in a Lrp4-independent manner, binding directly to the Fz-like domain of MuSK, which dimerizes and stimulates its phosphorylation. Preliminary experiments in C2 mouse muscle cells and WT mice confirmed that MuSK antibodies engaged with and stimulated MuSK and were well tolerated by the mice. A single IP injection of 2 mg/kg #13 antibody was sufficient to saturate MuSK at the synapse and increased its phosphorylation. Authors administered 10 mg/kg of the engineered antibody #13, or control human IgG1, by a single IP injection into male and female SOD1G93A mice after their disease onset (90 days of age). Twenty days after the single injection, they analyzed diaphragm muscle innervation and observed up to 2.7-fold increase in fully innervated synapses, and a 3.7-fold decrease of fully denervated synapses. Chronic administration with 10 mg/kg of a reverse chimera MuSK agonist antibody (murine IgG2A backbone lacking effector functions) every 24 days, decreased spinal cord motor neuron loss and maintained the number of innervated diaphragm neuromuscular synapses stable for 50 days more than control antibody, where neuromuscular junctions continue to denervate. Rescuing motor neurons and synaptic junctions resulted in an improved motor system output in #13 antibody treated mice, observed with the Compound Muscle Action Potential (CMAP) test. Treatment also extended lifespan of SOD1G93A mice by 7 days in females and 10 days in males (Cantor et al., 2018).

In 2019, Sengupta-Ghosh et al. (2019) tested MuSK agonist antibody #13 (on murine IgG2A backbone) by chronic IP administration of 10 or 100 mg/kg of antibody every 2 weeks in male and female SOD1G93A mice. Unlike Cantor et al. (2018), treatment started at 6 weeks of age, prior to disease onset, until 18 weeks of age (for histology analysis), or 22 weeks of age (for the functional analysis). Authors showed that treatment was able to preserve innervation of the NMJ in the diaphragm, as previously demonstrated. However, they discovered that it was insufficient to provide any functional benefit such as preservation of respiratory or diaphragm function (as measured by plethysmography or the CMAP test) nor was it able to alter the disease progression. Moreover, no improvement of motor neuron loss in the spinal cord and brainstem, no reduction of body weight loss, and no extension of mice survival were observed (Sengupta-Ghosh et al., 2019).

### Antibody-Based Interventions for IL-6 Receptor

Sporadic ALS patients show chronic peripheral and central nervous system inflammation characterized by infiltration in the spinal cord of inflammatory macrophages, IL-17A-positive T cells, and mast cells (Henkel et al., 2004; Fiala et al., 2010). Studies show that inflammatory macrophages play an important role in motor neuron death in the ALS conditions. Indeed, 19% of motor neurons in post-mortem ALS spinal cords, both healthy-appearing and apoptotic neurons, exhibit evidence of phagocytosis by interleukin-6 (IL-6)- and tumor necrosis factor-α (TNFα)-positive macrophages (Liu et al., 2012). Moreover, IL-6 signaling has proved toxic for mouse brain in a dose-dependent manner (Campbell et al., 2014).

Out of 31 studies included, 2 studies focused on the IL-6 receptor (IL-6R). These studies were published between 2006 and 2013. Both used a recombinant humanized monoclonal antibody named Tocilizumab.

In 2012, Mizwicki et al. provided evidence for attenuation of inflammatory activation in the peripheral blood mononuclear cells (PBMCs) and in the CSF of ALS patients through inhibition of IL-6R by treatment with Tocilizumab, a humanized antibody (HuAb) against IL-6R (Mizwicki et al., 2012). In 2013, Fiala et al. studied the *in vivo* effects of Tocilizumab infusion therapy in sporadic ALS patients (Fiala et al., 2013). Eleven patients with sporadic ALS (mean age 55 years) were selected for the study. Ten patients provided peripheral blood specimens. First, authors measured baseline mRNA expression of genes implicated in inflammation by RT-PCR and divided the patients according to the level of inflammation into group 1 (5 patients with high inflammation) and group 2 (5 patients with low inflammation) (Fiala et al., 2010; Mizwicki et al., 2012). Antibody infusion was performed every 4 weeks from 4 to 8 months, with doses increasing from 4 to 8 mg/kg. Blood samples were taken before and 1 h after each infusion. Levels of inflammatory genes were measured in blood PBMC (peripheral blood mononuclear cells) together with serum cytokines and compared to the previously analyzed baseline levels. Results showed that Tocilizumab infusions downregulated inflammatory genes (in particular IL-1 and IL-6) both in PBMC and serum of group 1 (high inflammatory baseline) whereas group 2 patients (low inflammatory baseline) experienced an up-regulation of IL-1 and IL-6 mRNAs levels together with a strong release of IL-6 in serum. Interestingly, the progression of neurological decline (measured by ALSFRS-R) was attenuated in three patients that adhered to the therapy. Although the study was not a controlled double-blind trial, authors were able to conclude that infusions of Tocilizumab might be beneficial for sALS patients by normalizing IL-1 and IL-6 expression, but that the effects are individual as well as time- and dose-dependent (Fiala et al., 2013).

In 2016, Lam et al. investigated the effect of Tocilizumab in rat primary cortical neurons exposed to an ALS patients' PBMC secreted proteins. The investigation of the antibody treatment was part of a bigger study on the transcriptional and epigenetic differences in PBMCs from two twins, one of whom being diagnosed for ALS (Lam et al., 2016). Although no genetic differences were found in hematopoietic cells of these twins, authors reported a higher abundance of CD14-positive monocytes and lower presence of T- and NK-cells in the ALS-twin compared to her healthy sibling. RNA sequencing of PBMCs supported these findings and highlighted significant changes in chemokines and metalloproteases genes produced by ALS PBMC. Moreover, PBMC form the ALS-twin spontaneously produced high concentrations of IL-6, IL-1 and TNF-α, whereas the PBMC of the healthy twin had the same levels only after stimulation with SOD1. Having observed a higher production of IL-6, authors treated cultured rat primary cortical neurons with PBMC supernatants for 6 h (1:100 ratio of supernatant to medium) and demonstrated that PBMC supernatant from the ALS-twin, but not that of the healthy twin, was toxic for neurons and increased their degeneration. The level of neuronal degeneration and death was strongly reduced by treating cells with Tocilizumab for 2 h, but not with anti-IL-1 or anti-TNF-α antibodies (Lam et al., 2016).

### Antibody-Based Interventions for Other Proteins

Finally, out of the 31 manuscripts analyzed, 8 papers described antibody interventions directed against other proteins, namely NRP-1, myostatin, CD40L, DR-6, IFN-γ, GD1a, CTGF and HMGB1. These studies were published between 2006 and 2019 and all used monoclonal antibodies. Four of them used mouse monoclonal Ab, and the other four used human or humanized monoclonal Ab. All these antibodies were tested in SOD1G93A mice and administered by IP injections, except for α-IFN-γ, which was administered ICV.

#### Neuropilin-1

Semaphorin 3A (Sema3A), a destabilizing factor for axonal innervation of neuromuscular junction, triggers distal axonopathy and muscle denervation through its receptor, Neuropilin-1 (NRP-1). Both proteins were upregulated in pre-symptomatic ALS mouse models and ALS patients (Körner et al., 2016; Maimon et al., 2018).

In 2014, Venkova et al. tested a humanized monoclonal antibody specific for the Sema3A docking CUB domains (a1a2) of NRP-1 (α-NRP-1A), provided by Genentech Inc (Venkova et al., 2014). In immortalized motoneuron-like cultured cells, α-NRP-1A antibody prevented Sema3A-induced growth cone collapse. In mice, α-NRP-1 antibody was administered by IP injections twice a week until end stage. When administered to pre-symptomatic (40 days of age) SOD1G93A mice, α-NRP-1A delayed and temporarily reversed motor functional decline, as assessed by rotarod task, and prolonged the lifespan. Moreover, it also reduced NMJ denervation and increased percentage of large-caliber axons in ventral roots. Despite these protective effects in pre-symptomatic treated mice, when administered at advanced stages of the disease (90 days of age) the antibody did not show any significant improvement (Venkova et al., 2014).

#### Myostatin

Myostatin is a member of the TGF-β superfamily. It regulates muscle development and homeostasis, by inhibiting both the proliferation and differentiation of satellite cells and myoblasts (McCroskery et al., 2003), and negatively regulates muscle regeneration after injury (McCroskery et al., 2005).

In 2006, Holzbauer et al. tested a myostatin/GDF-8 neutralizing antibody (RK35), previously generated from hybridoma cells obtained from mice immunized with recombinant myostatin (Whittemore et al., 2003), in both mouse and rat models expressing SOD1G93A (Holzbaur et al., 2006). The antibody was administered weekly, starting at pre-symptomatic stage (28 days of age) until sacrifice or end stage of the disease. In both mouse and rat models, inhibition of myostatin resulted in increased muscle mass and strength, which was maintained during the early stages (56–90 days of age) of disease but lost by end-stage. Myostatin inhibition slowed down degenerative changes in skeletal muscles (limb and diaphragm muscles) but did not delay the appearance of gait abnormalities or limb paralysis, nor did it extend the survival of diseased mice or rats. Changes observed in early stages were lost in late stages of the disease, except for the increase in diaphragm muscle mass and function that was maintained until the end stage in RK35-treated animals (Holzbaur et al., 2006).

#### CD40 Ligand

T-cell infiltration and activation has been observed during the disease progression in central and peripheral nervous system tissues of SOD1G93A mice (Lincecum et al., 2010). This phenomenon is mediated by the engagement of CD40L with its receptor (Roy et al., 1993).

In 2010, Lincecum et al. investigated the effect of a mouse monoclonal antibody, named MR1, directed against the mouse CD40 ligand (Lincecum et al., 2010). The antibody was derived from mouse hybridoma cells obtained after immunization of mice with IP injections of activated ThO (D1.6) (Noelle et al., 1992a,b). MR1R1 antibody was administered weekly starting at pre-symptomatic stage (50 days of age) until end stage. Treatment slowed weight loss, delayed disease onset, and extended survival of mice. Moreover, it lowered glial fibrillary acidic protein (GFAP) and Mac-2 staining, two markers glia activation, and reduced the loss of motor neurons (Lincecum et al., 2010).

#### Death Receptor 6

Death Receptor 6 (DR-6) is a tumor necrosis factor receptor which, after oligomerization, activates different pathways that can lead to neuronal death (Nikolaev et al., 2009).

In 2013 Huang et al. observed elevated levels of DR-6 in spinal cords of SOD1G93A mice and post-mortem tissues of ALS patients and investigated the effect of an anti-DR-6 antibody named 5D10 (Huang et al., 2013). The antibody was obtained from mouse hybridoma cells derived from mice after immunization with the recombinant protein. *In vitro*, the antibody promoted motor neuron survival, *via* activation of Akt phosphorylation and inhibition of the caspase 3 signaling pathways, following sodium arsenite treatment, growth factor withdrawal, or co-culture with SOD1G93A astrocytes. *In vivo*, IP delivery twice a week of 5D10 starting from asymptomatic stage (42 days of age) until 140 days of age promoted motor neuron survival, decreased astrocytes activation, and induced oligodendrocyte proliferation in the spinal cord and protected NMJ in gastrocnemius muscle from denervation. Moreover, 5D10-treated mice showed decreased pNfH levels in serum and 79% increase in hindlimb grip strength, suggesting improved motor function (Huang et al., 2013).

#### Interferon Gamma

IFN-γ is a pro-inflammatory cytokine whose levels were found increased in spinal cord of ALS mice and patients (Aebischer et al., 2011).

Otsmane et al. tested a rat IgG1 antibody against mouse IFN-γ, named R4-6A2, purified from hybridoma cell lines (Otsmane et al., 2014). *In vitro*, treatment with α-IFN-γ protected cultured mouse motor neurons from IFN-γ-induced death. The antibody was then delivered in the CSF of SOD1G93A mice by osmotic pumps ICV implanted, starting at early-onset of disease (13 weeks of age). In treated mice, authors observed a significant increase of surviving motor neurons but no changes in mice survival. The beneficial effect was observed only for the period during which the antibody was present in the CNS (Otsmane et al., 2014).

#### G1a and GT1b Gangliosides Complex

Gangliosides are glycosphingolipids that play important roles in the modulation of membrane proteins and ion channels, in cell signaling and in communication among cells (Sipione et al., 2020). GD1a and GT1b are enriched on the membrane of neurons and facilitate the interactions between oligodendrocytes and axons, promoting long-term axonal stability. Changes in the ganglioside profile have been observed in ALS patients and animal models (Rapport et al., 1985; Dodge et al., 2015).

Xu et al. tested a recombinant natural neurite-promoting human IgM antibody, named rHIgM12, that binds to the gangliosides complex GD1a and GT1b on the surface of neurons. In primary neurons, rHIgM12 promotes the cytoskeleton dynamics by decreasing tubulin acetylation and increasing its tyrosination (Xu et al., 2015). A single IP injection at pre-symptomatic stage (60 days of age) allowed the antibody to cross the blood brain barrier and reach the CNS. Authors reported increased survival in SOD1G93A and SOD1G86R mice (10 and 8 days, respectively), a 16-day delay in the onset of neurological deficits, as measured by nocturnal activity, and a 5-day delay of weight loss. Moreover, mice treated with rHIgM12 displayed fewer degenerating spinal cord axons and more NeuN-positive anterior horn neurons. Multiple doses of rHIgM12 in animal models were not effective due to the development of a strong anti-human antibody response that inactivated circulating rHIgM12 (Xu et al., 2015).

#### Connective Tissue Growth Factor (CTGF)

Connective tissue growth factor (CTGF/CCN2) is a matricellular protein, found up-regulated in spinal cord tissues of ALS patients (Spliet et al., 2003) and in muscles of symptomatic SOD1G93A mice (Gonzalez et al., 2017). Its involvement in ALS is still unclear although its functions in skeletal muscles (Morales et al., 2011) might be relevant for the pathology.

In 2018, Gonzalez et al. tested a human monoclonal IgG antibody against CTGF/CCN2, named FG-3019, obtained from FibroGen. Inc. (Gonzalez et al., 2018). The antibody was administered by IP injection to male mice at pre-symptomatic stage (8 weeks old), three times a week for 2 months. FG-3019 antibody delayed body weight loss and improved muscle function and locomotor capacity, as shown by longer hanging time, increased muscle strength and longer traveled distances at faster speed. Antibody reduced fibrosis and improved skeletal muscle architecture as demonstrated by reduced interstitial space and myofiber atrophy. Moreover it reduced NMJ denervation and myelin degeneration in the sciatic nerve (Gonzalez et al., 2018).

#### High Motility Group Box 1

High motility group box 1 (HMGB1) is an ubiquitously expressed nuclear protein but also an extracellular mediator of RAGE and TLR4 activation, two proteins implicated in the neuroinflammatory pathways observed in ALS (Brites and Vaz, 2014; Lee et al., 2015). Interestingly, HMGB1 has been shown to translocate from the nucleus to cytoplasm in ALS patients and animal models (Lo Coco et al., 2007; Casula et al., 2011), suggesting a potential pathogenic role for HMGB1 in ALS.

In 2019 Lee et al. tested a humanized IgG2b antibody targeting the extracellular damage-associated molecular pattern (DAMP) form of HMGB1 (Lee et al., 2019). The anti-HMGB1 (clone 2G7), purified from hybridomas, was administered to SOD1G93A female mice every week until end stage of disease. Administration started from 35 days of age (pre-onset) for prevention or from 70 days (post-onset) for treatment. Pre-onset treatment transiently improved muscle strength assessed by grip test and reduced key pro-inflammatory genes in spinal cord, whereas post-onset treatment had no effect. Treatment at none of the two time points extended the survival time nor did it affect neuroinflammation (Lee et al., 2019).

## Discussion

Antibody-based therapies are gaining relevance for the treatment of neurodegenerative diseases (Mortada et al., 2021) and the FDA approval of a passive immunization approach for Alzheimer's disease demonstrates the feasibility of these interventions for neurodegenerative conditions. According to our results, over the last 15 years, 31 original papers have been published demonstrating the potential of antibody-treatments for ALS.

The antibody-based strategies target both intracellular and extracellular proteins demonstrating the adaptability of immunoglobulins for therapeutic purposes. More than fifty percent of the revised paper indeed have been generated against extracellular proteins or membrane receptors with the aim of inhibiting the downstream effects of receptor activation. Interestingly, the other portion targeted intracellular proteins, known to cause ALS either in their pathological (misfolded or mutant SOD1, C9ORF72 repeats) or physiological (TDP-43) forms. Targeting a toxic intracellular protein as SOD1, TDP-43 or C9ORF72 repeats in ALS is an essential requirement, as these proteins are presently the main triggers of the pathology. Full-length antibodies possess two main advantages as therapeutic intracellular strategies. First, they can be specifically up-taken by neurons and microglial cells through the clathrin-dependent Fcγ receptor endocytosis (Peress et al., 1993; Congdon et al., 2013), two cell populations relevant to the disease; and second, once inside the cells, they activate physiological processes that induce the clearance of targeted proteins such as the TRIM-21/proteasome (McEwan et al., 2017) or the endosomes/lysosomes degradative machinery (Gu et al., 2013). On the other hand, the presence of the Fc domain may facilitate the activation of inflammatory processes, which can be avoided by using full-length-derived single chain antibodies or modified Fc domains, as demonstrated by some of the analyzed approaches.

Antibodies are big molecules and might pose difficulties in penetrating the CNS due to the natural defense structure of the blood-brain barrier (BBB). The use of single chain antibodies could overcome this issue since they are smaller in size and possess higher cellular penetration capacity. Moreover, scFv antibodies have the potential to enhance the target protein degradation, and the fact that they can be easily engineered increases their future therapeutic potential. On the other hand, it is worth mentioning that the delivery of a single chain antibody through viral vectors might present further limitations in controlling and modulating the treatment in case of adverse effects. Despite the limitations posed by BBB, most of the antibody-based interventions identified through the present systematic literature review used a systemic delivery of the immunoglobulin with efficient target engagement and therapeutic effect. The use of a systemic rather than localized delivery represents a huge advantage for patients since therapeutic interventions localized in the CNS might be riskier and more dangerous for them. That being said, systemic delivery might increase the risk of side effects as some of the targeted proteins are expressed also outside the CNS. This subject has been poorly investigated in the reviewed papers but appear necessary to characterize therapeutic interventions. Future studies should consider evaluating the presence and effect of antibodies delivered systemically also outside the CNS. Moreover, novel strategies should be considered to improve the bioavailability of a systemically delivered Ab into the CNS. It is worth mentioning that no intervention analyzed has used local muscular delivery. The neuromuscular junction is a crucial structure in the context of ALS as it represents the contact surface between degenerating motor neurons and atrophic muscles. This synaptic structure appears to be underestimated as a delivery hub and future therapeutic interventions should also consider this administration strategy to target both motor neurons and muscle cells or molecules involved in NMJ degeneration.

Another relevant issue to point out following the analysis of the selected literature is the use of ALS models. Although not all targeting SOD1, most of the analyzed studies used the mutant SOD1 model. This model is so far the most utilized as it perfectly recapitulates the symptomatology and pathological events occurring in ALS but, unfortunately, it also presents some limitations. First, it is highly heterogeneous. The different number of transgene copies, the various levels of mutant protein expressed, or the genetic background of the animals (Bendotti et al., 2020) make the comparison of the obtained results and their reproducibility highly difficult. Second, it represents a very small portion of familial ALS cases therefore, its use can limit the observed efficiency of the therapeutic intervention to few forms of ALS excluding many other ALS cases. It is, therefore, necessary to implement protocols where the target and the therapeutic efficiency are studied in other familial-derived ALS models and to increase scientific efforts toward developing models for sporadic ALS that represent the 90% of all ALS cases.

Most of the analyzed papers describe preclinical trial or proof of principle studies in animal- or patients-derived cells. Although the antigen presence was validated also in ALS patient tissues, a relevant discrepancy emerges when considering that the antigen expression is evaluated in postmortem tissues and then targeted with a therapy in animal models at the pre-symptomatic stage. As sporadic ALS patients are diagnosed only years after the beginning of their symptoms, therapeutic strategies for ALS should be investigated with the use of animal models only after the onset of symptoms to have a more translational approach for patients. Moreover, as described by some of the mentioned papers, a treatment starting at the pre-symptomatic stage often proves less effective when delivered at the symptomatic stage, corroborating the need to evaluate therapeutic efficacy when the pathology has already manifested itself. For this reason, the identification of early and prognostic biomarkers in ALS patients may give rise to new therapeutic strategies with a more precise intervention window and a better evaluation of the therapeutic efficacy.

## Conclusions

In conclusion, with this review we systematically searched and described scientific efforts made in the past years in the production and evaluation of therapeutic interventions with passive immunization in patients and models of amyotrophic lateral sclerosis. Although some improvements still need to be made in order to increase the efficiency in ALS patients, the therapeutic interventions analyzed in this review showcase the strong potential of antibodies as a therapeutic approach for ALS. Owing to the specificity of these molecules and their potential to block/inhibit downstream pathological events or increase the clearance of the pathogenic proteins, passive immunization is on its way to become a therapeutic intervention for this disease.

## Data Availability Statement

The original contributions presented in the study are included in the article/Supplementary Material, further inquiries can be directed to the corresponding author/s.

## Author Contributions

SP and AP-B designed the study. AP-B and ER performed the literature research and analysis. AP-B, ER, and SP wrote the manuscript. All authors listed have made a substantial, direct, and intellectual contribution to the work and approved it for publication.

## Funding

This work has been funded by the ALS Canada and Vincent Bourque Foundations. SP is the recipient of the 2020 ALS Career Transition Award from ALS Canada and Vincent Bourque Foundations and of the 2019 Marlene Reimer Brain Star of the year award CIHR-INMHA (ICT-171454).

## Conflict of Interest

The authors declare that the research was conducted in the absence of any commercial or financial relationships that could be construed as a potential conflict of interest.

## Publisher's Note

All claims expressed in this article are solely those of the authors and do not necessarily represent those of their affiliated organizations, or those of the publisher, the editors and the reviewers. Any product that may be evaluated in this article, or claim that may be made by its manufacturer, is not guaranteed or endorsed by the publisher.

## Abbreviations

a.a.: amino acids
AAV: adeno-associated virus
Ab: antibody
Abs: antibodies
AChRs: acetylcholine receptors
AEs: adverse events
ALS: amyotrophic lateral sclerosis
ASO: antisense oligonucleotides
BBB: blood-brain barrier
C9ORF72: chromosome 9 open reading frame 72
CD40: cluster of differentiation 40
CMAP: compound muscle action potential
CNS: central nervous system
CSF: cerebrospinal fluid
CTGF: connective tissue growth factor
DPRs: dipeptide repeat proteins
DR-6: death receptor 6
ECM: extracellular matrix
EDL: extensor digitorum longus
Fab: fragment antigen-binding
fALS: familial ALS
Fc: Fragment crystallizable
FDA: Food and Drug Administration
FRS-R: Functional Rating Scale-Revised
FTLD: frontotemporal lobar degeneration
GFAP: glial fibrillary acidic protein
GFP: green fluorescent protein
HEK: human embryonic kidney
HMGB1: high motility group box 1
HSP: heat shock protein
IC: intracortical
ICV: intracerebroventricular
IgG/M: Immunoglobulin G/M
IHC: immunohistochemistry
IL-6: interleukin 6
IN: intranasal
INF-γ: interferon gamma
IP: intraperitoneal
IT: intrathecal
IV: intravenous
LPS: lipopolysaccharide
MAb: monoclonal antibody
mMAb: mouse monoclonal antibody
MMT: manual muscle testing
MN: motor neurons
mSOD1: misfolded SOD1 protein
MuSK: muscle-specific kinase
NFκB: nuclear factor-kappa B
NK: natural killer
NMJ: neuromuscular junctions
Nogo: neurite outgrowth inhibitor
NRP-1: neuropilin-1
PBMC: peripheral blood mononuclear cell
PK: pharmacokinetics
RD: repeated dose
RRM1: RNA recognition motif 1
RT-PCR: reverse transcriptase polymerase chain reaction
SAEs: serious adverse events
sALS: sporadic ALS
scFv: single-chain variable fragment
SD: single dose
Sema3A: semaphorin 3A
SOD1: superoxide dismutase 1
SVC: slow vital capacity
TDP-43: TAR DNA-binding protein 43
TGF-β: transforming growth factor beta
TNF-α: tumor necrosis factor α
TRIM-21: tripartite motif containing-21
WT: wild type.
